# Community Knowledge of and Attitudes towards COVID-19 Prevention Techniques in Saudi Arabia: A Cross-Sectional Study

**DOI:** 10.3390/ijerph182312783

**Published:** 2021-12-03

**Authors:** Amal Khalil AbuAlhommos, Fatimah Essa Alhadab, May Mohammed Almajhad, Rahmah Almutawaa, Sara Taleb Alabdulkareem

**Affiliations:** Pharmacy Practice Department, Clinical Pharmacy College, King Faisal University, Hofuf 31982, Saudi Arabia; fatimessa08@gmail.com (F.E.A.); may24426@gmail.com (M.M.A.); r1_r2_@hotmail.com (R.A.); talebsara4@gmail.com (S.T.A.)

**Keywords:** attitudes, community, COVID-19, knowledge, prevention

## Abstract

The purpose of the study was to assess the community knowledge of and to obtain a broad overview of attitudes towards appropriate prevention techniques that are recommended by the Ministry of Health to prevent COVID-19 transmission in Saudi Arabia. Methods: A cross-sectional study using an online survey was conducted in Saudi Arabia between 1 May and 30 November 2020 to assess the community knowledge of and attitudes towards appropriate COVID-19 prevention techniques in Saudi Arabia. The study tool was developed based on an extensive literature review. Results: A total of 577 individuals were involved in this study. The majority of the participants knew that COVID-19 is classified as a severe acute respiratory syndrome, is caused by viral infection, and that it is more common among the elderly and those who have a chronic illness. More than half of the participants were able to identify the symptoms of COVID-19 correctly, which are fever, dry cough, and loss of taste. Approximately half the study participants were knowledgeable about appropriate distancing, handwashing, and preventive measures (e.g., wearing a cloth mask, smoking cessation, avoiding dangerous cultural behaviors that increase the probability of disease transmission). More than half of the study participants were able to identify the appropriate actions that should be taken if common COVID-19 symptoms appear. Conclusion: A promising level of knowledge and positive attitudes towards COVID-19 was observed in Saudi Arabia. Continuous efforts should be maintained to sustain the level of awareness among the public. Further studies are warranted to explore the level of knowledge and attitudes after the introduction of COVID-19 vaccines.

## 1. Introduction

Coronavirus disease 2019 (COVID-19), an infectious disease that emerged in China, is caused by a virus from the family *Coronaviridae,* which comprises positive-stranded RNA viruses. The family can be divided into four genera: *Alphacoronavirus*, *Betacoronavirus*, *Deltacoronavirus*, and *Gammacoronavirus*. In December 2019, in Wuhan City, China, scientists discovered a betacoronavirus that causes severe acute respiratory syndrome, coronavirus 2 (SARS-CoV-2), which is similar to a virus found in bats. It can be transmitted by close contact between people through the respiratory droplets of an infected person [1,2,3,4,5,6]. Additionally, literature findings show that SARS-CoV-2 aerosol transmission is possible [7].

The life cycle of the virus within the host consists of five steps: The first step is attachment, when the virus binds to the receptors of the host. The second step, penetration, is when the virus enters cells by endocytosis. The third step is biosynthesis, whereby the RNA of the virus enters the nucleus of cells and starts replicating. The fourth step is maturation, in which the viral content is made. Finally, in the fifth step, the viral content is released. Coronaviruses contain different proteins, such as envelope, nucleocapsid, spike, and membrane. The spike protein in SARS-CoV-2 binds to ACE2 receptors in the host, which are highly expressed in the lung, kidneys, and heart. It enters lung epithelial cells and destroys them, causing lung injury. In response to this injury, macrophages start fighting this virus and T cells also act in response to this virus [8]. Mutation is defined as a change in the genetic sequence [9]. Genomes that differ from each other in genetic sequence are called variants. Variants can differ from each other by one or more mutations. When a phenotypic difference is demonstrated among the variants, they are called strains [10]. The SARS-CoV-2 genome is subject to multiple mutations that lead to antigenic drift which can lead to escape from immune recognition. Different variants have been identified during the pandemic in different countries. The most important three, which have rapidly become dominant, are the UK variant (Alpha variant), the South African variant (Beta variant), and the Brazilian variant (Gamma variant) [11].

The most important factors that can exacerbate the spread of COVID-19 include travelling to an area that has a high risk of transmission, older age (patients older than 50 years), having comorbidities such as cardiovascular disease, hypertension, diabetes, respiratory disease, smoking, liver disease, cancer, organ transplant, and immunosuppression, and pregnancy [4]. The clinical presentations of patients who are suspected of having COVID-19 are fever, cough, dyspnoea, loss of the sense of smell and taste, fatigue, sputum production, sore throat, neurological symptoms, nasal congestion, chest pain, tachypnea, tachycardia, and cyanosis. Certain diagnostic tests should be conducted in patients suspected of having COVID-19. These tests include reverse transcription-polymerase chain reaction (RT-PCR) tests, arterial blood gas (ABG) tests, full blood count (FBC), serum interleukin-6 level test, chest x-rays, computed tomography (CT) test, lung ultrasound, and SARS-CoV-2 nucleic acid testing [8].

We can prevent the transmission and the spread of COVID-19 in different ways. Firstly, we can practice hand hygiene by washing our hands with soap and water or using an alcohol-based hand rub. Social distancing is also encouraged, whereby individuals should maintain a distance from each other of at least one meter, which is equal to three feet, to prevent the transmission of infected droplets through sneezing and coughing. People should stay away from crowded areas to prevent contact with an infected person. When people leave the house, they should practice respiratory hygiene by using a tissue while sneezing or coughing, or by covering their mouth and nose with a bent elbow. Following this, people should wash their hands and throw the tissue away. If a person has any symptoms, he/she should isolate themselves at home until these symptoms disappear, and if they must leave the house, they should wear a mask to prevent spreading the virus to other people. If they have a fever, difficulty breathing, and cough, they should contact a doctor and seek medical attention. Covering the mouth and nose with a mask is mandatory. Surgical masks and N95 respirators are recommended for healthcare workers [12]. While previous studies in Saudi Arabia have focused on the clinical and mental consequences of COVID-19 [13,14,15,16,17], very few studies have explored community knowledge and attitudes towards the disease, and most of them are restricted to a specific governorate or area. Therefore, this study aimed to assess the community knowledge of and to obtain a broad overview of attitudes towards the appropriate prevention techniques that are recommended by the Ministry of Health (MOH) to prevent COVID-19 transmission in Saudi Arabia before the emergence of COVID-19 drift variants.

## 2. Methods

### 2.1. Study Design

A cross-sectional study using an online survey was conducted in Saudi Arabia between 1 May and 30 November 2020 to assess the community knowledge of and attitudes towards appropriate COVID-19 prevention techniques in Saudi Arabia.

### 2.2. Sampling Strategy

Eligible participants were invited to participate in the study using a convenience sampling technique. The general population was invited to participate in the study through social media platforms (Facebook and WhatsApp). General Facebook and WhatsApp groups were utilized to distribute the study invitation letter, together with the survey participation link. These included groups that discuss customer services, healthy food, and restaurants and cafes, which include a large number of members with different socio-demographic characteristics.

All participants voluntarily participated in the study and were thus considered exempt from written informed consent. The survey was distributed through dedicated pages/groups in the social media platforms (Facebook and WhatsApp). The survey distribution post requested the participants to participate in the study and share the post with their friends and network. The study aims and objectives were clearly explained at the beginning of the survey. The inclusion criteria were participants aged 18 years and above, currently living in Saudi Arabia. Participants were excluded if they were below 18 years of age.

### 2.3. Study Tool

The study tool was developed based on an extensive literature review. It comprised three sections. The first section, which addressed the participants’ demographics, comprised five questions about age, gender, nationality, education level, and employment status. The second section of the questionnaire addressed participants’ knowledge about COVID-19 (16 questions), including its symptoms, methods of transmission, and prevention techniques. The third section of the questionnaire was composed of 11 questions and addressed participants’ attitudes towards the appropriate prevention techniques that are recommended by MOH, Appendix A.

### 2.4. Sample Size

Using a confidence interval of 95%, a standard deviation of 0.5, and a margin of error of 5%, the required sample size was 385 participants.

### 2.5. Statistical Analysis

Descriptive statistics were used to describe participants’ demographic characteristics. Continuous data were reported as a mean ± SD for normally distributed variables. Categorical data were reported as percentages (frequencies). A two-tailed *p* < 0.05 was considered statistically significant. The statistical analyses were carried out using SPSS (version 25).

## 3. Results

A total of 577 individuals were involved in this study. The majority (77.1%, *n* = 445) were females. Approximately 46.0% were aged 18–29 years. Approximately half (56.8%, *n* = 328) had a bachelor’s degree. Approximately 35.7% (*n* = 206) were without a job. For further details on the characteristics of the study participants, refer to Table 1.

### 3.1. Knowledge about COVID-19

Table 2 below highlights participants’ knowledge about COVID-19. The Ministry of Health was the main source of information for approximately two-thirds (68.3%) of the study participants. Approximately, 84.7% (*n* = 489) of the participants were aware that COVID-19 is classified as a severe acute respiratory syndrome. A majority of 97.4% (*n* = 562) of the participants knew that it was discovered initially in Wuhan, China. The same percentage of the participants knew that it causes viral infection. The majority of the participants identified that direct contact with an infected person, respiratory droplets, and touching contaminated surfaces are common causes of COVID-19 transmission. Approximately 62.0% of the participants were able to identify the symptoms of COVID-19 correctly, which are fever, dry cough, and loss of taste. The vast majority (96.2%, *n* = 555) of the participants knew that severe illness with COVID-19 is more common among the elderly and those who have a chronic illness. Approximately half of the participants (49.0%) knew that a distance between individuals of two to three meters is the method for the prevention of COVID-19 recommended by the MOH. Approximately half of the participants (48.0%) acknowledged that there is treatment for the disease. Approximately 40.0% of the participants were able to identify the appropriate duration for handwashing (40 s) and when using alcohol for hand hygiene (20 s). The majority (85.1%, *n* = 491) were able to identify that wearing a cloth mask is an appropriate procedure to decrease the risk of infection; however, only 4.5% of the participants were able to identify that smoking cessation decreases the risk of getting infected with the disease. Only one-third of the study participants were able to identify the proper steps for handwashing. Approximately 75% were able to identify the best method after sneezing and coughing to reduce transmission of COVID-19, which is covering the mouth and nose with a tissue and then washing one’s hands. Additionally, the vast majority (84.6%) were able to identify conditions that require patients to isolate at home.

### 3.2. Attitudes towards COVID-19

Table 3 below highlights participants’ attitudes towards COVID-19. Approximately 60.0% of the study participants reported that they stay at home to reduce the spread of the virus most days during the week. Additionally, 36.0% reported that they go out only in case of emergency. Only 11.4% of the participants reported that they have attended gatherings that include more than 50 persons. The vast majority (86.8%) reported that they avoid cultural behaviors such as shaking hands.

The most common situations in which the study participants reported washing their hands with water and soap were after sneezing and coughing, before and after taking care of an infected person at home, and after using the toilet. Approximately 72.0% of the participants were able to identify the proper procedure if common COVID-19 symptoms appear, which is wearing a face mask, gloves, and seeking prompt medical care. In the case of visiting a clinic and receiving a negative result, the majority (75.9%) reported that they would adhere to the preventive measures that were advised by the MOH. If the result was negative and there had been previous contact with a confirmed COVID-19 case, approximately 60.0% reported that they would quarantine at home for 14 days.

In the case of visiting a clinic and receiving a positive result without symptoms, with no previous contact with a confirmed COVID-19 case, approximately one-third (35.7%) reported that they would adhere to home quarantine for 14 days. If the result was positive and they had severe symptoms, approximately two-thirds (69.1%) reported that they would seek emergency medical assistance. In the case of testing positive for COVID-19 without the option of isolating at home, approximately 40.0% reported that they would go to a hotel.

## 4. Discussion

This study aimed to assess the community knowledge of and attitude towards the appropriate prevention techniques that are recommended by MOH to prevent COVID-19 transmission in Saudi Arabia. The main findings of this study are: (1) the majority of the participants knew that COVID-19 is classified as a severe acute respiratory syndrome, causes viral infection, and that it is more common among the elderly and those who have a chronic illness; (2) more than half of the participants were able to identify the symptoms of COVID-19 correctly, which are fever, dry cough, and loss of taste; (3) approximately half the study participants were knowledgeable about the appropriate distancing, handwashing, and preventive measures (wearing a cloth mask, smoking cessation, avoiding dangerous cultural behaviors that increase the probability of disease transmission), and (4) more than half of the study participants were able to identify the appropriate actions that should be taken if common COVID-19 symptoms appear.

Overall, the participants in our study showed a very good level of knowledge regarding COVID-19 disease in terms of its symptoms, methods of transmission, and prevention techniques. This confirms the findings of previous studies on the level of knowledge among the general public in Saudi Arabia concerning previous epidemics, such as the Middle East respiratory syndrome (MERS) [18,19].

Proper knowledge about COVID-19 disease, its symptoms, methods of transmission, and prevention techniques are key factors that have contributed to the improvement in the epidemiological status of COVID-19 in Saudi Arabia. This is reflected positively in the number of daily new cases, active cases, and daily new deaths [20]. As of November 26, 2021, 98% of COVID-19 cases were recovered/discharged, with only 2,049 active cases (with a prevalence rate of 0.006%) in the whole country (the total population is 34.81 million) [20].

The majority of the participants in our study (84.7%) were aware that COVID-19 is classified as a severe acute respiratory syndrome and causes viral infection (97.4%). Most participants identified that direct contact with an infected person, respiratory droplets, and touching contaminated surfaces are common causes of COVID-19 transmission. Approximately 62.0% of the participants were able to identify the symptoms of COVID-19 correctly, which are fever, dry cough, and loss of taste. The vast majority (96.2%) of the participants knew that severe illness with COVID-19 is more common among the elderly and those who have a chronic illness. Multiple factors might have contributed to the high level of knowledge about COVID-19 among our study participants. These include the high level of education and the young population [21,22,23]; in our study, more than half of our study population reported that they have a bachelor’s degree or higher and more than one-third had a high-school education. A good level of knowledge has proven to be a predictor for adherence to precautionary measures during pandemics [24,25]. On the other hand, a lack of awareness and not having enough information about the disease leads to undesirable attitudes and practices, which ultimately leads to negative consequences for infection control [26].

Wearing a face mask and taking other precautionary measures are important actions that should be taken by the whole community and specifically by those who are sick or have a higher risk of getting infected [27,28]. Besides, there is a continuous need to follow social distancing to prevent the spread of the coronavirus.

In our study, more than half of the participants (68.3%) reported that the MOH was the main source of information about COVID-19 followed by social media (22.0%). Confirming the findings of a previous study that was conducted in Egypt, the MOH was one of the main sources of information to the general public about COVID-19 through its communication via different media platforms, including television, social media (Facebook), advertisements in the street, and mobile messages [21,29]. Communication should be clear and target the whole community taking into account individual differences [30,31]. A previous study highlighted that one of the main motivations of the general public to follow the guidance of the MOH regarding COVID-19 disease was to protect themselves and their family from getting infected with the disease [21]. The governmental authorities in Saudi Arabia have a great deal of experience in dealing with public health issues such as the COVID-19 pandemic. Two previous viral disease outbreaks that emerged in Saudi Arabia in the past decade, SARS and MERS [32,33,34,35,36], were managed with the highest possible healthcare standards that led to suppressing their spread and diminishing their clinical and economic impact on the general public.

In our study, the participants showed a positive attitude towards appropriate prevention techniques. Approximately 60.0% of the study participants reported that they stay at home to reduce the spread of the virus most days during the week, while 36.0% reported that they go out only in the event of emergency. Only 11.4% of the participants reported that they have attended celebrations that included more than 50 people. The vast majority (86.8%) reported that they avoid certain cultural behaviors, such as shaking hands. A positive attitude could be justified due to the proactive protective measures taken by policymakers in Saudi Arabia, which has helped markedly in controlling the spread of the disease, despite the large population in Saudi Arabia and the heavy traffic of visitors who travel to Saudi Arabia for religious purposes. Preventive measures have included suspending religious visits to the country (Umrah), lockdown, and restrictions on international and domestic flights, public gatherings, and face-to-face education. This positive attitude of the general public reflects the trust in the government’s actions in controlling the pandemic, which confirms the findings of previous studies [21,22,37].

In our study, most participants held positive attitudes towards controlling the spread of COVID-19, which was reflected in their practices. This was demonstrated by the precautionary measures that were reported, including practicing proper hygiene and avoiding crowded places. Approximately half of the participants (49.0%) knew that maintaining a distance of two to three meters from other people is the recommended method for the prevention of COVID-19 by the MOH. Approximately 40.0% of the participants were able to identify the appropriate duration for handwashing (40 s) and when using alcohol for hygiene (20 s). The majority (85.1%) were able to identify that wearing a cloth mask is an appropriate procedure to decrease the risk of infection; however, only 4.5% of the participants were able to identify that smoking cessation decreases the risk of becoming infected with the disease. Only one-third of the study participants were able to identify the proper steps for handwashing. Approximately 75% were able to identify the best method after sneezing and coughing to reduce transmission of COVID-19, which is covering the mouth and nose with a tissue and then washing one’s hands.

Approximately 72.0% of the participants were able to identify the proper procedure if common COVID-19 symptoms appear, which is wearing a face mask, gloves, and seeking prompt medical assistance. More than half of the study participants were able to identify the appropriate actions that should be taken if common COVID-19 symptoms appear. These include adherence to the preventive measures as advised by the MOH, quarantining at home for 14 days, and seeking emergency medical assistance in the case of severe symptoms. This confirmed the findings of previous studies that were conducted in Saudi Arabia and Egypt, highlighting the positive attitude of the general public [21,29]. This positive attitude has emerged from the general belief of the community in the effectiveness of the preventive measures that are recommended by the MOH, the WHO, and other leading bodies in healthcare in the prevention of the spread of the disease, which was reflected clearly in the epidemiological curves of the country [38]. These positive attitudes and practices towards controlling the spread of COVID-19 and preventing its transmission are reflections of the preventive measures taken by the authorities in Saudi Arabia. Similar findings were found across different studies conducted in China and Malaysia [37,39].

Policymakers in governments are advised to continue their efforts in increasing the level of awareness regarding COVID-19 prevention and transmission in society. These efforts should be directed towards high-risk populations, including populations with lower socioeconomic status [40].

This study has limitations. The use of social media platforms to recruit participants could affect the socioeconomic and demographic characteristics (i.e., having a higher proportion of respondents from one gender over the other or including a younger population) of the study participants, which may result in bias and could affect the generalizability of the study findings. Our study excluded participants who were aged below 18 years, therefore, we might have omitted an important group of the community who might have a different level of knowledge and attitudes towards COVID-19. The use of an online survey for data collection could have missed some of the targeted population. However, this has been common research practice during the pandemic as a previous study has documented that the use of online evaluation platforms during the COVID-19 pandemic has increased significantly and specifically for education and research purposes [41]. However, due to the COVID-19 pandemic and in order to minimize the risk of disease transmission, we preferred not to undertake any direct (in person) recruitment and to use social media platforms instead. A further consideration is that the data collection extended over a period of six months, which could have led to changes in the level of knowledge over the time period. Finally, some questions that explored participants’ attitudes towards COVID-19 used a yes/no format. This type of question might affect the participants’ responses and lead to social desirability bias; the participants might provide a socially desired response regarding their own behavior and attitudes towards COVID-19.

## 5. Conclusions

Our study participants showed an encouraging level of knowledge and positive attitudes towards COVID-19. Continuous efforts should be maintained to sustain the level of awareness among the public. Further studies are warranted to explore the level of knowledge and attitudes after the introduction of COVID-19 vaccines. A study of the intermediate period (i.e., late 2021) would be useful to look at progression in awareness as vaccination programs develop. Additionally, future studies to show progressive changes in attitudes during the recovery period would be valuable.

## Figures and Tables

**Table 1:** Participants’ demographic characteristics.

Demographic Variable	Frequency (%)
Age category	
18–29 years	265 (45.9)
30–39 years	160 (27.7)
40–49 years	116 (20.1)
50–59 years	30 (5.2)
60 years and above	6 (1.0)
Gender	
Female	445 (77.1)
Nationality	
Saudi	570 (98.8)
Education level	
Primary education	5 (0.9)
High school level	222 (38.5)
Bachelor degree	328 (56.8)
Post graduate degree	22 (3.8)
Employment status	
Without job	206 (35.7)
Student	160 (27.7)
Retired	21 (3.6)
Has a job outside the medical field	145 (25.1)
Has a job in the medical field	45 (7.8)

**Table 2:** Participants’ knowledge about COVID-19.

Variable	Frequency (%)
What is Coronavirus disease (COVID-19)?	
Severe Acute Respiratory Syndrome	489 (84.7)
Seasonal Influenza	51 (8.8)
Common Cold disease	7 (1.2)
I don’t know	30 (5.2)
Where COVID-19 was discovered initially?	
In Wuhan, China	562 (97.4)
In the United States	4 (0.7)
In Saudi Arabia	2 (0.3)
I don’t know	9 (1.6)
COVID-19 is considered as?	
Viral infection	562 (97.4)
Bacterial infection	10 (1.7)
I don’t know	5 (0.9)
COVID-19 can be transmitted by (you can choose more than one option)?	
Direct contact with infected person	491 (85.1)
Direct person droplet	475 (82.3)
Touching contaminated surfaces	458 (79.4)
Blood transfusion	100 (17.3)
Stools	25 (4.3)
I don’t know	1 (0.1)
COVID-19 symptoms are (you can choose more than one option)?	
Fever, dry cough, and loss of taste	357 (61.9)
Fever, dry cough, and shortness of breath	217 (37.6)
Fever, vomiting, and skin rash	1 (0.2)
I don’t know	2 (0.3)
Covid-19 more likely to be severe in?	
Elderly and have chronic illnesses	555 (96.2)
Children	9 (1.6)
Elderly and have no chronic illnesses	5 (0.9)
Pregnant women	1 (0.2)
What are the prevention methods that were recommended by MOH?	
Distance between you and your partner 2 to 3 m	283 (49.0)
Stay at home to help reduce spread of virus	174 (30.2)
Avoid crowded area more than 25 person	83 (14.4)
Clean hand for 2 min	35 (6.1)
I don’t know	2 (0.3)
Did you know if we have any curable therapy	
Yes	279 (48.4)
How many seconds should we take in washing our hand?	
20 s	214 (37.1)
25 s	20 (3.5)
30 s	115 (19.9)
40 s	228 (39.5)
Did you use alcohol hygiene, if yes how long?	
10 s	223 (38.6)
20 s	221 (38.3)
30 s	64 (11.1)
40 s	69 (12.0)
Do you think any one of the following procedures may reduce the risk of infection by COVID-19?	
Wearing cloth masks	491 (85.1)
Taking herbal remedies	36 (6.2)
Smoking cessation	26 (4.5)
Taking of antibiotics	24 (4.2)
In the steps of washing hands we should consider:	
Wet hands, rub palms together, interlink your fingers, rub palms with your fingers, rub the back of hands, then thoroughly rinse with water.	214 (37.1)
Wet hands, rub palms together, interlink your fingers, rub the back of hands, rub palms with your fingers, then thoroughly rinse with water.	178 (30.8)
Wet hands, rub palms together, rub the back of hands, interlink your fingers, rub palms with your fingers, then thoroughly rinse with water.	162 (28.1)
I don’t know	23 (4.0)
Best method after sneezing and coughing to reduce transmission of COVID-19:	
Cover mouth and nose with tissue and then washing hands	430 (74.5)
Cover mouth and nose with arm	107 (18.5)
Cover mouth and nose with tissue	38 (6.6)
I don’t know	2 (0.3)
The condition that is required for patients who need home isolation:	
Separate bathroom, staying in private room, Surface disinfection, especially door handles, open window frequently, use disposable materials that is for one use only.	488 (84.6)
Shared bathroom with the family, staying in a private room, disinfecting surfaces, especially door handles, opening the window frequently,	75 (13.0)
I don’t know	14 (2.4)
Do you think if there was vaccination to COVID-19:	
Yes	233 (40.4)
From where you got your information about COVID-19 prevention method:	
Ministry of Health	394 (68.3)
Social media	127 (22.0)
Internet website	43 (7.5)
From family and friends	13 (2.3)

**Table 3:** Participants’ attitudes towards COVID-19.

Variable	Frequency (%)
Did you stay at home to reduce the spread of virus most of the days during the week (5 days or more)?	
Yes	347 (60.1)
I go out in emergency case	207 (35.9)
No	23 (4.0)
Did you attend any celebration site that include more than 50 persons?	
Yes	66 (11.4)
Did you keep the distance (1 to 2 m) between you and others to avoid spread of COVID-19?	
Yes	501 (86.8)
Have you recently avoided cultural behaviors, such as shaking hands?	
Yes	496 (86.0)
Did you wash your hand by water and soap after following situation?	
After sneezing and coughing	435 (75.4)
Before and after take care of infected person in home	424 (73.5)
After leaving the toilet	389 (67.4)
Before meal	94 (16.3)
After touching the garbage	42 (7.3)
After touching animals (pets)	36 (6.2)
After leaving home	13 (2.3)
I don’t do	2 (0.3)
If COVID-19 common symptoms appear, patient must do the following:	
Wear face mask, wear gloves and contact the emergency	416 (72.1)
Wear face shield, Wear face mask and stay at home	109 (18.9)
Wear face mask and stay at home	41 (7.1)
Wear face mask and wear gloves	11 (1.9)
In case of you visit clinic and your result was negative, what you will do?	
Commitment to preventive precautions that applied by MOH	438 (75.9)
Home quarantine for 14 days	89 (15.4)
Home isolation for 10 days	45 (7.8)
Contact the emergency	5 (0.9)
In case of you visit clinic and your result was negative, and you are in previous contact with COVID-19 confirmed case. What you will do?	
Home quarantine for 14 days	347 (60.1)
Home isolation for 10 days	116 (20.1)
Commitment to preventive precautions that applied by MOH	64 (11.1)
Contact the emergency	50 (8.7)
In case of you visit clinic and your result was positive without symptoms, and you aren’t in previous contact with COVID-19 confirmed case. What you will do?	
Home isolation for 10 days	223 (38.6)
Home quarantine for 14 days	206 (35.7)
Commitment to preventive precautions that applied by MOH	120 (20.8)
Contact the emergency	28 (4.9)
In case of you visit clinic and your result was positive with severe symptoms. What you will do?	
Contact the emergency	347 (69.1)
Home quarantine for 14 days	136 (23.6)
Commitment to preventive precautions that applied by MOH	61 (10.6)
Home isolation for 10 days	33 (5.7)
In the case of positive COVID-19 Patient and there is no place for isolation in his house, what he most does:	
Go to hotels	231 (40.0)
Contact the emergency	189 (32.8)
Take precautions at home	139 (24.1)
I don’t know	18 (3.1)

## Data Availability

Not applicable.

## Appendix A

Questionnaire tool

Assess Community knowledge of and attitudes towards COVID-19 prevention techniques in Saudi Arabia: a cross-sectional study.

Dear participant,

You are kindly requested to participate in our study which is aiming to assess the community knowledge of and get a broad overview of attitudes towards the appropriate prevention techniques that are recommended by the MOH to prevent COVID-19 transmission in Saudi Arabia.

Answer the following questions (choose one answer)

Demographic characteristics

1. **Gender**
  ○ Male
  ○ female
2. **Age**
  ○ 18–29
  ○ 30–39
  ○ 40–49
  ○ 50–59
  ○ Over 60
3. **Nationality**
  ○ Saudi
  ○ Non-Saudi
4. **Education level**
  ○ Uneducated
  ○ High school or less
  ○ Bachelor’s degree
  ○ Post graduate degree
5. **Employment status**
  ○ Without job
  ○ Retired
  ○ Has a job in non-medical field
  ○ Has a job in medical field

**Awareness about COVID-19 disease**

6. **what is Coronavirus disease (COVID-19)**
  ○ Seasonal Influenza
  ○ Common Cold disease
  ○ Severe Acute Respiratory Syndrome
  ○ I don’t know
7. **where COVID-19 was discovered initially**
  ○ in United State
  ○ in Saudi Arabia
  ○ in Egypt
  ○ in Wuhan city, China
  ○ I don’t know
8. **COVID-19 consider as**
  ○ virus infection
  ○ bacterial infection
  ○ fungal infection
  ○ I don’t know
9. **COVID-19 can be transmitted by (you can choose more than one answer)**
  ○ Touching contaminated surfaces
  ○ Direct contact with infected person
  ○ Direct person droplet
  ○ Blood transfusion
  ○ Stools
  ○ I don’t know
10. **COVID-19 symptoms are (you can choose more than one answer)**
  ○ Fever, dry cough, shortness of breath
  ○ Fever, dry cough, loss of taste
  ○ Fever, vomiting, skin rash
  ○ I don’t know
11. **COVID-19 more likely to be severe in**
  ○ children
  ○ elderly and have chronic illnesses
  ○ elderly and have no chronic illnesses
  ○ pregnant women
  ○ I don’t know

**Attitude towards COVID-19 disease**

12. **What are the prevention methods that were recommended by MOH**
  ○ Stay at home to help reduce spread of virus
  ○ Distance between you and your partner 2 to 3 m
  ○ Avoid crowded area more than 25 person
  ○ Clean hands for 2 min

**Answer the following question by yes or no**

13. **Did you stay at home to reduce the spread of virus most of the days during the week** **(5 days or more)**
  ○ Yes
  ○ No
  ○ I go out in emergency case
14. **Did you attend any celebration site that include more than 50 persons?**
  ○ Yes
  ○ No
15. **Did you keep the distance (1 to 2 m) between you and others to avoid spread of COVID-19**
  ○ Yes
  ○ No
16. **Have you recently avoided cultural behaviors, such as shaking hands?**
  ○ Yes
  ○ No
17. **Did you know if we have any curable therapy**
  ○ Yes
  ○ No
  ○ I don’t know
18. **Did you wash your hand by water and soap after following situation**
  ○ After leaving the toilet
  ○ Before and after take care of infected person in home
  ○ After sneezing and coughing
  ○ After touching animals (pets)
  ○ After touching the garbage
  ○ Before meal
  ○ After leaving home
  ○ I don’t do
19. **How many seconds should we take in washing our hand?**
  ○ 20 s
  ○ 30 s
  ○ 40 s
  ○ 25 s
20. **Did you use alcohol hygiene if yes how long**
  ○ 20 s
  ○ 40 s
  ○ 30 s
  ○ 10 s
21. **Do you think any one of the following procedures may reduce the risk of infection by COVID-19**
  ○ Taking of antibiotics
  ○ Taking herbal remedies
  ○ Wearing cloth masks
  ○ smoking cessation
22. **In the steps of washing hands we should consider.**
  ○ Wet hands, rub palms together, rub the back of hands, interlink your fingers, rub palms with your fingers, then **thoroughly rinse** with water.
  ○ Wet hands, rub palms together, interlink your fingers, rub the back of hands, rub palms with your fingers, then **thoroughly rinse** with water.
  ○ Wet hands, rub palms together, interlink your fingers, rub palms with your fingers, rub the back of hands, then **thoroughly rinse** with water.
  ○ I don’t know
23. **Best method after sneezing and coughing to reduce transmission of COVID-19**
  ○ Cover mouth and nose with arm
  ○ Cover mouth and nose with tissue
  ○ Cover mouth and nose with tissue and then washing hands
  ○ I don’t know
24. **If COVID-19 common symptoms appear, patient most do the following:**
  ○ Wear face mask, wear gloves, wear face shield
  ○ wear face shield, Wear face mask and stay at home
  ○ Wear face mask, wear gloves and contact the emergency 937
  ○ Wear face mask, wear gloves and stay at home
25. **what you will do in the following situations:**

**In case of you visit fever clinic and your result was negative, what you will do?**
  ○ Commitment to preventive precautions that applied by MOH
  ○ Home isolation for 10 days
  ○ Home quarantine for 14 days
  ○ call 937
**In case of you visit fever clinic and your result was negative, and you are in previous contact with COVID-19 confirmed case. What you will do?**
  ○ Commitment to preventive precautions that applied by MOH
  ○ Home isolation for 10 days
  ○ Home quarantine for 14 days
  ○ call 937
**In case of you visit fever clinic and your result was positive without symptoms, and you aren’t in previous contact with COVID-19 confirmed case. What you will do?**
  ○ Commitment to preventive precautions that applied by MOH
  ○ Home isolation for 10 days
  ○ Home quarantine for 14 days
  ○ call 937
**In case of you visit fever clinic and your result was positive with severe symptoms. What you will do?**
  ○ Commitment to preventive precautions that applied by MOH
  ○ Home quarantine for 10 days
  ○ Home quarantine for 14 days
  ○ call 937

26. **The condition that is required for patients who need home isolation:**
  ○ Separate bathroom, staying in private room, Surface disinfection, especially door handles, open window frequently, use disposable materials that is for one use only.
  ○ Private room, surface disinfect.
  Private room, open window frequency.
  ○ I don’t know
27. **Do you think if there was vaccination to COVID-19:**
  ○ Yes
  ○ No
  ○ I don’t know
28. **Do you think one of this condition can decrease the possibility of getting infected with COVID-19:**
  ○ Using of antibiotics.
  ○ Using herbal medication and products
  ○ Using face mask
  ○ Smoking cessation
29. **From where you got your information about COVID-19 prevention method:**
  ○ Internet website
  ○ Social media
  ○ Ministry of Health
  ○ From family and friends
30. **In the case of positive COVID-19 Patient and there is no place for isolation in his house, what he most does:**
  ○ Go to hotels
  ○ Contact the emergency
  ○ Take precautions at home
  ○ I do not know
